# Malathion-induced hematotoxicity, hepatotoxicity, and nephrotoxicity in male chicks

**DOI:** 10.3389/fvets.2026.1765548

**Published:** 2026-02-13

**Authors:** Imad Mohamed Tahir Fadlalla, Khalid Musa Ali, Badr Hasab-Elrasoul Aljack, Muzzamil Atta

**Affiliations:** 1 Department of Genetic Research, IRMC, Imam Abdulrahman Bin Faisal University, Dammam, Saudi Arabia; 2 Department of Biomedical Sciences, College of Veterinary Medicine, Sudan University of Science and Technology, Khartoum, Sudan; 3 Department of Poultry Sciences, College of Animal Production, Sudan University of Science and Technology, Khartoum, Sudan; 4 Department of Animal Resources, Ministry of Municipality and Environment, Doha, Qatar

**Keywords:** chickens, hematotoxicity, hepatotoxicity, malathion, nephrotoxicity

## Abstract

**Introduction:**

Exposure to malathion has been linked to various toxicities that can affect nearly every organ in the human body. This study aimed to examine how malathion toxicity affects certain sero-biochemical and hematological parameters in chickens in order to determine the harmful effects on the blood, liver, and kidneys.

**Methods:**

Two experiments were conducted as part of the study design. Experiments 1 and 2 were used to determine the LD50 value and the hazardous dose in chickens, respectively. In the first experiment, 10 groups of 30 birds each were used. Over a period of 4 weeks, each group was divided into five subgroups (with six birds in each replicate), and one group served as a control. In the second experiment, six groups of birds and one control group of 10 chicks each were used (60 + 10 birds).

**Results:**

The LD_50_ in this study was found to be 620 mg/kg of body weight. As the hazardous dose of malathion increased, the levels of hemoglobin (Hb) and packed cell volume (PCV) gradually decreased. Compared to the control group, all treatments had significantly higher red blood cells (RBC) and total leukocyte counts (TLC) (*p* < 0.01). Albumin and glucose concentrations increased in tandem with the increases in malathion dosages. Significant increases were observed in aspartate aminotransferase (AST), alkaline phosphatase (ALP), gamma-glutamyl-transferase (GGT), and alanine aminotransferase (ALT) levels. Bilirubin, creatinine, and urea levels progressively increased when dosages were increased. Triglycerides (TG) and total cholesterol (TC) levels exhibited modest decreases as dosages increased. Toxic levels of malathion had a severe impact on magnesium and inorganic phosphate levels.

**Conclusion:**

Hepatic, renal, and hematological functions are sensitive markers of malathion toxicity. The findings of the study provide a fundamental basis for understanding the acute toxicity of malathion in chickens. This study provides useful information for regulatory monitoring and risk assessment.

## Introduction

Malathion, a commonly used organophosphate (OP), is a major contributor to the burden of insecticide poisonings, which constitute a substantial global public health concern (1). Pesticide residues in marketed poultry meat and its products pose a major health risk due to their occupationally hazardous nature. Therefore, one of the most crucial elements in reducing potential risks to human health is monitoring pesticide residues in poultry meat (2). Many pesticides remain in food and feed sources, making them dangerous environmental contaminants. Globally, the usage of pesticides increased by 180%, particularly in developing countries (3). Approximately 32–44% of all pesticides derived from organophosphorus are malathion (S-1,2-bis(ethoxycarbonyl)ethyl O, O-dimethyl phosphorodithioate). Malathion is used to manage mosquitoes and other insects. However, malathion pollution arises from its uncontrolled usage, leaving residues in the soil, natural water systems, and food products (4, 5). According to several studies (6, 7), malathion causes significant harm to the kidneys, liver, and other essential tissues. According to Amira (8), malathion exposure has been linked to several toxicities that affect almost every organ in the human body.

There have also been reports regarding the harmful effects of malathion on the liver, kidneys, testis, ovaries, lungs, pancreas, and blood. Body weight, feed conversion ratio, and feed intake all decreased as the dose of malathion increased during acute and chronic risk assessments. Furthermore, the chicks’ liver and cold carcass weights decreased (9). Martinez and Leyhe (10) state that malathion decomposes into malaoxon, which is more toxic than the parent compound. A toxic form of malathion is produced by cytochrome P450 enzymes, which convert malathion to malaoxon. Malaoxon is toxic because it inhibits acetylcholinesterase (11).

ElGaddal et al. (12) report that malathion was first used in Sudan in 1962 as part of the Gezira and Managil project to manage cotton pests and in campaigns to eradicate malaria. Sudan utilizes 121 tons of malathion annually (13). In the chicken industry, malathion is widely used as a detergent and to control external parasites such as lice, fleas, and mites (14).

By the end of the fourth week, malathion had considerably changed all hematological and biochemical parameters in the treatment group. The red blood cell (RBC) count, hemoglobin (Hb) concentration, packed cell volume (PCV), and leukocytes (WBCs) were all lower than those in the control group. Serum levels of alkaline phosphatase (ALP), alanine aminotransferase (ALT), aspartate aminotransferase (AST), lactate dehydrogenase (LDH), urea, creatinine, and uric acid were considerably higher in the malathion-treated group compared to the control group. Additionally, the serum levels of total protein, albumin, and globulin were significantly lower in the malathion-treated group compared to the control group. This finding explains the nephrotoxicity and hepatotoxicity associated with malathion (15).

Urinary malathion concentrations and both BChE activity assays showed significant inverse correlations, highlighting the dynamic link between exposure and enzymatic inhibition (1). By the end of the fourth week, the rats treated with malathion had significantly lower levels of triglycerides and very low-density lipoprotein (VLDL) and significantly higher levels of total cholesterol, alkaline phosphatase (ALP), alanine aminotransferase (ALT), aspartate aminotransferase (AST), and lactate dehydrogenase (LDH). Additionally, the rats treated with malathion had much lower levels of albumin and serum total protein (16). The study’s objectives were to standardize the method for determining the median lethal dosage (LD_50_) of malathion and investigate the toxicity of malathion in chickens in order to clarify its safety limits.

## Materials and methods

### Experimental design

A total of 370 clinically healthy male chickens were used in this study. The birds had approximately the same body weight (650–700 g) and were of the same age. Two weeks prior to treatment, the birds were housed under conditions with sufficient ventilation to allow for adaptation. Two experiments were designed, and the birds were randomly allocated to each experiment. In the first experiment, in order to determine the toxic dose and biochemical alterations caused by malathion, 300 male birds, aged 2 months, were divided into 10 groups, each containing 30 birds. Each group was further divided into five subgroups (with six birds in each replicate). The groups were divided into nine treated groups and one untreated control group based on the birds’ weights. In the second experiment, the lethal dose (LD_50_) of malathion (technical EC 57%) in chicks was determined by dividing 70 young male chickens into 6 groups and 1 control group of 10 chicks each (60 + 10 birds).

### Management

All the birds were reared until they were 2 months old and weighed between 650 and 700 grams, to be used for the treatment. The experimental conditions included a temperature range of 22–27 °C and a humidity range of 55–65%. Antibiotics, thiabendazole, and sulfamethazine were administered to the chickens to control roundworms, coccidia, and bacterial infections. Experimental birds were vaccinated against Newcastle and Gumboro diseases during the adaptation period (15 days). The birds were allowed to access feed and water ad libitum.

### Experiment 1

#### Determination of LD_50_ of malathion in male chickens

This experiment was conducted on clinically healthy male chickens of the same body weight and age, following the OECD guidelines (17). To determine the LD_50_, six experimental groups and one control group of ten chicks each (60 + 10 birds) were treated with successive doses. The doses were administered with an interval of 100 mg/kg body weight as a constant factor between every two successive doses beginning at 500 mg/kg body weight. The chicks were observed for 24 h, during which the number of dead chicks in each group and their symptoms were recorded. The LD_50_ was determined using the equation provided by Stewart and Stolman (18):


$$\;{\mathrm{LD}}_{50}=\text{the largest dose}-\frac{\sum (a\times b)}{n}$$

where:

a = the constant factor between two consecutive doses
b = the mean of the dead chicks in two successive groups
*n* = number of animals in the group
∑ = the sum of (a × b)

### Experiment 2

#### Determination of the toxic dose of malathion in male chickens

We investigated the toxic effects of malathion using the methodology outlined by Stewart and Stolman (18). Three hundred male chickens were used in this study. The experiment included 10 groups, each with 30 birds/treatment, and each treatment consisted of 5 subgroups (6 birds/replicate). Of this, 9 groups were treated groups, and the tenth group was the control group. The treated groups were intoxicated with 1/12 the LD_50_ of malathion for 28 days. The groups received various oral dosages, starting with a dose of 50 mg/kg body weight and continuing to increase the treatment dose at a rate of 50 mg/kg body weight as a constant factor between every two consecutive treatments. The chickens in the following groups received different doses of malathion: 50 mg/kg body weight for group (A), 100 mg/kg body weight for group (B), 150 mg/kg body weight for group C, 200 mg/kg body weight for group D, 250 mg/kg body weight for group E, 300 mg/kg body weight for group F, 350 mg/kg body weight for group G, 400 mg/kg body weight for group H, and 450 mg/kg body weight for group I.

#### Blood sample collection

Blood samples were collected from the cervical blood vessels into sterile, dry vacutainers (Becton-Dickinson) at the end of the 4th week of treatment. The serum was separated and stored at −20 °C until analysis. The serum was examined to evaluate the lipid profile, glucose levels, and liver and kidney function. To check for hematological indices, whole blood samples were collected into heparinized, sterile, dry vacutainers.

#### Hematological indices

The procedures followed were those outlined by Schalm (19). The following parameters were examined: hemoglobin concentration (Hb), packed cell volume (PVC%), count of red blood cells (RBCs), total leukocyte cells (TLC), and differential leukocyte count (DLC).

#### Assessment of liver functions

The concentration of total serum proteins (TP), albumin, bilirubin, alkaline phosphatase (ALP), alanine transaminase (ALT), gamma-glutamyl-transferase (GGT), and aspartate transaminase (AST) activity was determined using commercial kits (SPINREACT) according to the manufacturer’s instructions.

#### Determination of glucose level

Glucose levels were determined using commercial kits (SPINREACT) according to the manufacturer’s instructions.

#### Determination of lipid profile concentrations

Total cholesterol and triglyceride (TG) levels were measured using SPINREACT commercial kits (SPINREACT S.A.U., Girona, Spain), according to the manufacturer’s instructions.

#### Assessment of kidney functions

The spectrophotometric method was used to test blood urea and creatinine levels using commercial kits (SPINREACT), according to the manufacturer’s instructions.

#### Determination of serum macrominerals

Ca, P, and Mg serum levels were tested using an atomic absorption spectrophotometer (AA 6650 Shimadzu, Japan) and flame photometer (Corning, 400R; Essex, UK). Na and K levels were examined using commercial kits (Stanbio Laboratories, USA).

### Statistical analysis

An unpaired Student’s *t*-test was used to analyze differences between the mean values of toxic doses and blood serum parameters (44). The significance of the effect of treatment level (nine treatment levels plus a control) was evaluated using one-way analysis of variance (ANOVA) with *STATISTICA* software (StatSoft, Inc., OK, USA) (20). When significant differences were detected, mean comparisons were performed using Duncan’s multiple range test (DMRT) at *p* ≤ 0.05 as a *post-hoc* test. The median lethal dose (LD₅₀) was determined according to the equation described by Stewart and Stolman (18).

## Results

This study examined the effects of toxic dosages of malathion on healthy male chickens of the same age and body weight in terms of hemotoxicity, hepatotoxicity, and nephrotoxicity. In this study, the LD_50_ of malathion in chicks was 620 mg/kg body weight. Single oral doses of 250, 300, 350, 400, and 450 mg/kg of malathion cause toxic symptoms in chickens, which culminate in death between the third and fourteenth days.

### Hematological parameters

The effects of different malathion dosages on specific hematological markers in chickens are shown in Table 1. As the toxic dose of malathion increased, the Hb and PCV levels gradually decreased. According to the findings, changes in RBC and total leukocyte count (TLC) were considerably larger (*p* < 0.01) for all treatments during the malathion treatment period compared to the control group. Although TLC dropped in the 400 and 450 mg/kg body weight groups, it was still greater than in the control. The mean TLC differential counts obtained during malathion poisoning are shown in Table 1. Compared to the control group, monocyte and neutrophil counts were significantly increased (*p* < 0.01). The differences between basophils and eosinophils after malathion poisoning were not statistically significant (*p* < 0.05). Eosinophil counts have not changed significantly, but basophil counts have clearly increased.

**Table 1:** Hematological parameter associated with different toxic doses of malathion on male chickens.

Parameter	Dose-mg									Control	SE	*p* value
	50	100	150	200	250	300	350	400	450			
Hb Count mg/dl	19.26^b^	18.12^c^	17.24^c^	14.74^d^	8.02^e^	7.44^ef^	6.90^fg^	6.80^fg^	6.22^g^	26.70^a^	0.343	**
RBC Count ×(10)^6^	36.8^e^	36.6^e^	36.0^e^	39.0^cd^	38.2^d^	40.2^bc^	41.0^b^	41.4^b^	45.6^a^	29.8^f^	0.473	**
PCV (%)	24.6^c^	22.0^d^	19.8^e^	20.4^de^	17.4^f^	15.8^fg^	14.6^g^	12.8^h^	28.0^b^	31.0^a^	0.569	**
TLC Count ×(10)^3^	110.6^f^	112.6^f^	132.2^d^	134.8^d^	140.0^c^	190.6a	180.2^b^	119.2^e^	87.6^g^	68.4^h^	1.037	**
Neut.	2.0	2.0	2.0	2.0	3.0	3.0	3.0	3.0	3.0	1.0	0.000	**
Eosin.	1.0	1.0	1.0	1.0	1.0	1.0	1.0	1.0	1.0	1.0	0.000	NS
Basoph.	12.0^e^	14.0^d^	16.0^c^	16.4^c^	22.0^b^	22.2^b^	22.0^b^	24.2^a^	23.0^b^	7.4^f^	0.365	**
Monocy.	1.0^g^	12.6^e^	15.8^d^	17.0^cd^	17.8^c^	18.2^bc^	19.4^b^	21.8^a^	21.0^a^	3.6^f^	0.468	**

** = the treatment effect is significant (*p* < 0.05). NS = the treatment effect is not significant. a, b, c, d, e, f, g = means on the same row of different superscripts are significantly different (*p* < 0.05).

### Renal function tests

Renal function parameters were examined by measuring serum creatinine and urea concentrations. Renal functions are obviously impacted by malathion toxicity, which depends on the dosage and duration of treatment. Compared to the control, the urea and creatinine concentrations were elevated (Table 2). As shown in Table 2, salt concentration dropped in patients receiving low dosages, but it was unaffected in those receiving high doses (300–450 mg). Low dosages of malathion had no effect on potassium levels, whereas those who received doses of 250 mg and 540 mg saw a drop. As the toxic dose of malathion increased, the calcium concentration increased significantly in all groups, with the exception of those treated with a dose of 450 mg, which showed a slight drop compared to the control. Toxic dosages had a severe impact on magnesium and inorganic phosphate, which were dramatically reduced in all treated groups.

**Table 2:** Renal function parameter associated with different toxic doses of malathion on male chickens.

Parameter	Dose									Control	SE	Sign. level
	50	100	150	200	250	300	350	400	450			
Urea mg/dl	11.08^e^	11.92^e^	17.5^de^	17.18^e^	17.54^de^	22.74^cd^	30.48^b^	22.98^c^	41.58^a^	10.24^e^	1.762	**
Creatinine mg/dl	0.82^c^	0.87^c^	1.30^bc^	1.44^b^	1.46^b^	2.30^a^	2.20^a^	2.40^a^	2.58^a^	1.00^bc^	0.170	**
Sodium	168.0^bc^	157.4^cd^	150.2^d^	119.6^e^	132.4^e^	183.8^ab^	174.6^abc^	190.6^a^	172.6^abc^	172.6^abc^	5.799	**
Potassium	12.8^ab^	11.72^ab^	13.22^a^	13.1^de^	13.44^e^	10.76^b^	10.60^b^	6.08^cd^	8.08^c^	12.84^ab^	0.757	**
Calcium	9.28^cd^	8.68^d^	12.30^cd^	9.64^cd^	11.70^cd^	16.76^ab^	17.78^ab^	20.74^a^	13.68^bc^	9.88^cd^	1.467	**
Phosphate	7.18^b^	6.18^c^	4.36^d^	1.64^f^	1.80^f^	2.57^e^	2.36^ef^	1.83^f^	1.82^f^	10.52^a^	0.230	**
Magnesium	2.22^cd^	2.50^c^	1.82^d^	3.76^b^	3.28^b^	1.81^d^	1.92^d^	2.16^cd^	1.96^cd^	5.26^a^	0.177	**

** = the treatment effect is significant (*p* < 0.05). a, b, c, d, e, f, g = means on the same row of different superscripts are significantly different (*p* < 0.05).

### Liver functions and glucose parameters

A drop in the albumin fraction is often linked to a decrease in blood total protein when assessing liver function. Table 3 shows that all treatment groups exhibited a noticeably greater increase in bilirubin levels than the control group. The liver enzyme levels of AST, ALT, ALP, and GGT steadily increased with increasing malathion doses compared to the control group, as shown in Table 3. In this study, the toxic dose was gradually increased to elevate glucose levels in all treatment groups.

**Table 3:** Liver function parameter associated with different toxic doses of malathion on male chickens.

Parameter	Dose-mg									Control	SE	*p* value
	50	100	150	200	250	300	350	400	450			
Total protein g/dl	3.36b^c^	3.48^b^	2.84^bc^	2.66^c^	2.70^bc^	2.64^c^	2.76^bc^	3.26^bc^	3.30^bc^	5.54^a^	0.245	**
Albumin- g/dl	2.94	3.48	2.94	2.40	2.32	2.40	1.22	1.20	1.46	2.70	0.321	**
Bilirubin- mg/dl	2.21	6.00	3.29	3.52	2.01	6.19	5.48	5.22	7.92	0.561	0.871	**
AST -U/L	72.62	62.81	75.54	180.48	236.29	244.11	307.39	338.42	457.87	158.02	15.821	**
ALT- U/L	9.59	21.72	16.55	19.80	24.21	25.28	31.85	25.80	30.66	13.06	2.678	**
ALP- U/L	1161.32	1114.56	1396.90	627.96	459.02	495.50	743.84	958.10	670.78	1178.78	76.113	**
GGT - U/L	18.13	25.66	17.02	18.57	19.86	28.94	19.24	22.29	32.78	12.23	1.882	**
Glucose -mg/dl	205.8^d^	229.0^cd^	243.8^c^	309.6^a^	320.2^a^	318.8^a^	315.2^a^	274.4^b^	277.0^b^	180.0^e^	8.917	**

** = the treatment effect is significant (*p* < 0.05). a, b, c, d, e, f, g = means on the same row of different superscripts are significantly different (*p* < 0.05).

### Lipid profile parameters

The lipid parameter indicators used in this study were total cholesterol (TC) and triglycerides (TG). As shown in Table 4, TC levels gradually increased as malathion dosages increased and were noticeably greater than those of the control group. Triglyceride (TG) levels were significantly decreased when compared to the control group.

**Table 4:** Lipid profile associated with different toxic doses of malathion on male chickens.

Parameter	Dose-mg									control	SE	*p* value
	50	100	150	200	250	300	350	400	450			
T. Cholesterol	142.62	151.60	164.96	204.94	225.50	311.12	315.50	222.24	182.76	171.46	2.175	**
Triglyceride (TG)	125.24	122.60	118.60	94.40	73.60	66.60	37.40	37.80	28.40	135.20	0.906	**

** = the treatment effect is significant (*p* < 0.05).

## Discussion

This study investigated the effects of malathion toxicity in male chicks on certain sero-biochemical and hematological parameters to ascertain its harmful effects on blood, liver, and kidney. This study indicated that the LD50 of malathion, which is toxic and lethal to chicks, was 620 mg/kg body weight and higher when administered as a single dose. According to Betty (21), all chicks died by the 19th day after being fed 5,000 parts per million of malathion. Asha (22) reported that in albino mice, the LD_50_ of malathion was 589 mg/kg body weight.

In this study, hematological changes produced by different malathion toxic doses compared to the control group showed that hemoglobin (Hb) and packed cell volume (PCV) levels decreased gradually with an increase in dose. According to Mrong et al. (23) and Rabab et al. (15), this could be explained by the significant cytotoxic effects of malathion pesticides. According to Srivastava et al. (24) and Subburaj et al. (25), hemoconcentration may be the primary cause of this decrease. Dehydration and heme ion losses in chicks are thought to be caused by diarrhea and water deficiency. According to Sagone et al. (26) an intrathoracic airway obstruction or pulmonary insufficiency that developed with exposure to a toxic dose is suggested by the gradual increase in the red blood cell (RBC) count in all treated groups, which increased the demand on the bone marrow for red blood cell production.

This study demonstrated that increasing the toxic doses of malathion led to a progressive increase in total leukocyte counts (TLC). Inflammation caused by the poisonous material resulted in considerably higher differential counts of neutrophils, monocytes, and basophils, whereas eosinophils did not approach significant values (*p* < 0.05). Abdul Rauf (27) reports that when exposed to malathion, the hematological changes included a large increase in neutrophil count and a significant drop in hematocrit and leukocyte count. According to Elzoghby et al. (15), rats treated with malathion had significantly lower levels of leukocytes (WBCs), packed cell volume (PCV), and hemoglobin concentration (Hb) than the control group.

In this study, all treatment groups showed a significant increase in the liver enzymes AST, ALT, and GGT. This study was consistent with research conducted by Ahmed et al. (28) and Abdel-Salam et al. (29).

In this study, bilirubin levels increased with an increase in the toxic dose of malathion. In severe toxic situations, bilirubin increased, resulting in hepatocellular dysfunction (30, 31). According to Medani and Adam (32) bilirubinemia linked to elevated ALP and GGT activity as well as elevated cholesterol content implies significant hepatic damage. All chicks treated with malathion had albumin and total protein levels. The balance between the processes of biosynthesis and catabolism, or loss due to hemorrhage or proteinuria, is represented by this decrease in the total protein concentration. According to Cornelius and Kaneko (33), a decrease in serum total protein concentration is typically linked to an increase in gamma globulin levels. These findings were consistent with research conducted by Kalender (16) and Flehi-Slim (34).

Additionally, malathion-intoxicated chicks had much lower triglyceride levels and significantly greater serum total cholesterol levels than the control group. These findings are consistent with those of Kalender (16) and Flehi-Slim (34). Increased serum lipid values, oxidative stress, inflammatory biomarkers, and liver and kidney damage are indicators of malathion-induced toxicity (35). All treatment groups in this study had higher glucose levels. Thus, the current study’s findings are corroborated by those of Bharti and Rasool (6) and Panahi et al. (36), which showed that malathion raises blood glucose levels by causing hyperglycemia through effects on skeletal muscle and hepatic glucose metabolism. The toxic dose of malathion affects the pancreatic enzymes essential for insulin secretion, such as glucokinase (GK) and glutamate dehydrogenase (GDH), which increase glucose levels.

This investigation demonstrated that all treated groups had significant increases in the levels of urea and creatinine, which were followed by an increase in the hazardous dose. An increase in urea and creatinine levels could be a sign of serious renal disease (33). According to a study by Bharti and Rasool (6), among the biochemical changes in malathion, higher urea levels were identified, and serum creatinine levels indicated renal dysfunction. Serum urea and creatinine levels were considerably higher in the malathion-treated group than in the control group, according to Khalid et al. (37) and Elzoghby et al. (15). A kidney biopsy in a case of acute kidney injury and nephrotic syndrome following malathion inhalation showed tubular cell damage, glomerular epithelial cell damage, and renal tissue necrosis (38, 39).

According to the findings of the study, sodium (Na) concentration marginally increased at high doses and decreased at low doses. The rapid development of clinical symptoms in chicks was likely the cause of the variation in sodium content. Malathion treatment had an impact on potassium levels, which dramatically decreased. According to Brouwer (40), alternate routes of excretion could be the cause of the drop in potassium levels. In this investigation, malathion had an impact on calcium concentration; high doses resulted in a notable increase in calcium concentration, similar to salt. This study found that all treated groups had lower phosphorus levels, with substantial reductions at high dosages. Since it is widely known that animals typically produce alkaline urine and that a significant amount of phosphates are expelled, it is not surprising to see a lower level of phosphate concentration in long-term treatment (41, 42). Magnesium concentrations decreased in all treatments, according to the current study. Ahmad (43) reports that fish exposed to malathion have higher levels of glutamate pyruvic transaminase (GPT), glutamate oxaloacetate transaminase (GOT), and alanine amino transferase (ALT). Although the impacts were negligible, calcium and magnesium ions were also impacted.

## Conclusion

Malathion is one of the organophosphorus substances that has been widely used since 1937. Determining the risk factors linked to malathion toxicity depends critically on the clinical importance of specific laboratory test results. This study aims to determine the effects of different malathion dosages on particular sero-biochemical and hematological traits in male chicks. The results showed that malathion significantly affected the majority of hematological and biochemical findings, depending on the dosage and duration of treatment. These results establish the detrimental impact of malathion on renal dysfunction and liver damage.

Recommendations for further research should be made in order to ascertain and control the use of malathion in light of its harmful risks and lethal consequences. The effects of malathion on various essential organs, such as the DNA damage, lung, heart, testis, and spleen, should be further investigated.

## Data Availability

The original contributions presented in the study are included in the article/supplementary material, further inquiries can be directed to the corresponding author.
